# Platelet-Neutrophil Interplay: Insights Into Neutrophil Extracellular Trap (NET)-Driven Coagulation in Infection

**DOI:** 10.3389/fcvm.2019.00085

**Published:** 2019-06-20

**Authors:** Amanda Z. Zucoloto, Craig N. Jenne

**Affiliations:** Department of Microbiology, Immunology and Infectious Diseases, Snyder Institute for Chronic Diseases, The University of Calgary, Calgary, AB, Canada

**Keywords:** platelets, neutrophils, neutrophil extracellular traps, NETs, coagulation, inflammation

## Abstract

Well established for their central role in hemostasis, platelets have increasingly been appreciated as immune cells in recent years. This emerging role should not come as a surprise as the central immune cells of invertebrates, hemocytes, are able to phagocytose, secrete soluble mediators and promote coagulation of hemolymph, blurring the line between immunity and hemostasis. The undeniable evolutionary link between coagulation and immunity becomes even clearer as the role of platelets in inflammation is better understood. Platelets exert a range of immune-related functions, many of which involve an intimate interplay with leukocytes. Platelets promote leukocyte recruitment via endothelial activation and can serve as “landing pads” for leukocytes, facilitating cellular adhesion in vascular beds devoid of classic adhesion molecules. Moreover, platelets enhance leukocyte function both through direct interactions and through release of soluble mediators. Among neutrophil-platelets interactions, the modulation of neutrophil extracellular traps (NETs) is of great interest. Platelets have been shown to induce NET formation; and, in turn, NET components further regulate platelet and neutrophil function. While NETs have been shown to ensnare and kill pathogens, they also initiate coagulation via thrombin activation. In fact, increased NET formation has been associated with hypercoagulability in septic patients as well as in chronic vascular disorders. This review will delve into current knowledge of platelet-neutrophil interactions, with a focus on NET-driven coagulation, in the context of infectious diseases. A better understanding of these mechanisms will shed a light on the therapeutic potential of uncoupling immunity and coagulation through targeting of NETs.

## Introduction

Poised at the interface of immunity and coagulation, platelets express a plethora of surface molecules and receptors and carry granules packed with hundreds of biologically active products. Platelets arise from megakaryocytes, which in turn differentiate from pluripotent hematopoietic cells restricted to the bone-proximal osteoblastic niche in the bone marrow (1, 2). Proplatelets are released from this specialized site into the circulation, continue to mature, ultimately releasing mature platelets (3).

Platelets are undoubtedly critical for hemostasis, in a large part by supporting blood coagulation. Upon activation, platelets expose negatively-charged phospholipids on the outer leaflet of their plasma membrane, providing an ideal surface for the assembly of coagulation factors complexes, such as the VIIIa-IXa complex and the Xa-Va complex. Moreover, factor XI and thrombin are brought into close proximity through interactions with the glycoprotein (GP) Ib/V/IX complex on the surface of platelets, facilitating factor XI cleavage by thrombin. This not only sustains the coagulation cascade, but also overcomes coagulation arrest in the presence of TFPI (inhibitor of the extrinsic pathway) (4).

Platelets are equipped with numerous immune receptors. For instance, signaling through TLRs 2/6 and 1/2 triggers platelet activation, marked by increased expression of surface CD62P (P-selectin), degranulation and aggregation (5–7). TLR4 engagement was shown to induce platelet aggregation and interaction with leukocytes as well as affect CD62P expression in a ligand-dependent manner (8–10). Moreover, human platelets have been shown to secrete antimicrobial peptides targeting both bacteria and fungi in response to thrombin, a key enzyme in the coagulation cascade (11). Platelets have also been increasingly recognized for their role in cellular recruitment. In that sense, platelets have been shown to serve as a “landing pad” in endothelial beds devoid of adhesion molecules, such as the brain (12, 13). In a model of hepatitis, CD8+ T cells were also shown to preferentially dock onto platelets adherent within liver sinusoids rather than adhering to endothelial cells themselves, suggesting a role for platelets in adaptive immunity (14).

The active role of platelets in both coagulation and immunity hints at an evolutionary link to the central immune cells of invertebrates, hemocytes. Hemocytes not only provide host defense through secretion of microbicidal peptides and phagocytosis, but also through coagulation of the hemolymph. In these invertebrate organisms, clot formation is a potent host defense mechanism as it isolates and contains the infectious agent (15–17). This ability to sequester and contain pathogens undeniably resembles the role of neutrophil extracellular traps (NETs), an immune effector mechanism of higher vertebrates. The complex interplay between platelets and neutrophils, which is most likely a long-evolving relationship, will be discussed in this review, with a focus on NET-driven coagulation.

## Platelet Dependent Neutrophil Recruitment and Activation

**Figure 1:**
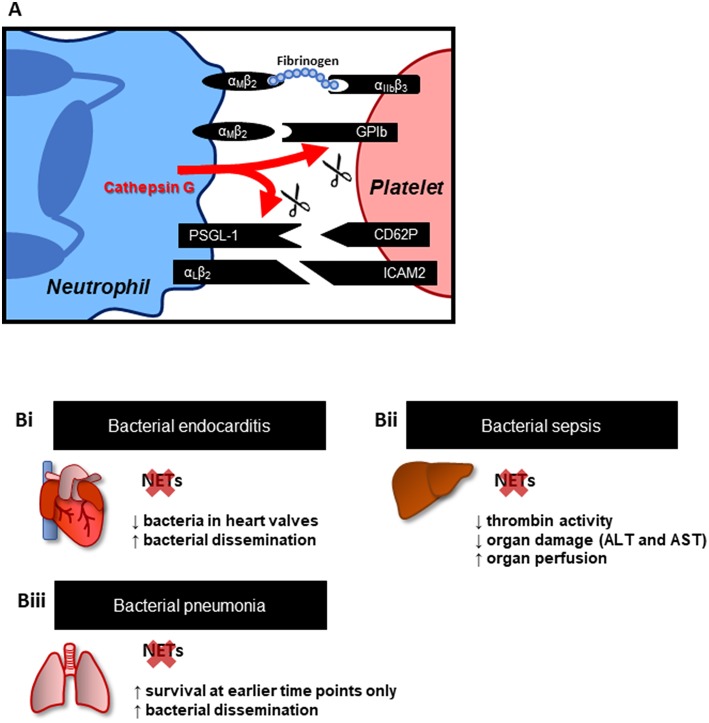
Platelets-neutrophil interactions and NETs. **(A)** Key adhesion molecules involved in platelet-neutrophil interactions. These interactions not only provide mechanisms of cell attachment but may also trigger intracellular signaling, promoting cell activation, resulting in the upregulation of additional adhesion and effector molecules. Effector molecules from both cells, such as cathepsin G from neutrophils, in turn modulate neutrophil-platelet physical interactions through cleavage of PSGL-1 and GPIb. **(B)** Effect of NET inhibition (PAD4^−/−^ mice) or disruption (DNase treatment) in animal models of **(Bi)** endocarditis (18), **(Bii)** bacterial sepsis (19), and **(Biii)** bacterial pneumonia (20). Overall, targeting NETs is associated with reduced inflammation and organ damage; however, this effect has been shown to favor bacterial dissemination.

The interactions between platelets and neutrophils are orchestrated by both their surface and secreted molecules, with the former allowing for physical interactions between these cells. Activated platelets express CD62P, which binds to P-selectin glycoprotein ligand-1 (PSGL-1) on the surface of neutrophils (Figure 1A) (21, 22). Alternatively, both GPIb or the integrin αIIβ3 on platelets interact with the integrin αMβ2 on leukocytes either directly, or through fibrinogen as a bridging molecule (23–25). Secreted molecules, such as cathepsin G produced by activated neutrophils, can disrupt these interactions through cleavage of GPIb and PSGL-1 (26). Moreover, there is increasing evidence of platelet-derived products modulating neutrophil recruitment, activation and function. For instance, CD40L secreted by platelets has been shown to upregulate integrin expression on neutrophils (27). Serotonin and CXCL4 have also been implicated in platelet-dependent neutrophil recruitment in models of abdominal inflammation and acute pancreatitis (28, 29).

Platelets mediate leukocyte recruitment via two main mechanisms: (a) by serving as a docking site for immune cells along the endothelium surrounding the inflammatory focus and (b) through secretion of chemoattractants. The extent to which platelets promote cellular recruitment appears to be tissue- and model-dependent. For instance, neutrophil infiltration into the peritoneal cavity, skin and brain in response to LPS was shown to be platelet-dependent whereas in the lung, a compensatory mechanism characterized by the upregulation of CXCL1 and CCL5 was able to overcome platelet depletion (30). In a model of *Pseudomonas aeruginosa* pulmonary infection, however, platelet depletion was shown to reduce neutrophil infiltration in the lung (31). Similarly, platelet-driven neutrophil recruitment to the colon and kidney has been demonstrated in models of dextran sodium sulfate (DSS)-induced acute colitis and cecal ligation and puncture (CLP) (32, 33). In these studies, platelet depletion was shown to improve clinical and histopathological scores, whereas in the aforementioned model of *P. aeruginosa* pulmonary infection, platelet depletion led to increased bacterial dissemination and mortality (31–33). These differences further emphasize the complexity of the interplay between platelets and other immune cells, such as neutrophils.

Indeed, platelets are not limited to providing a port-of-entry to neutrophils into sites of tissue insult. Platelets have been shown to directly stimulate the production of NETs through the process of NETosis (18, 34–37). In turn, NETs amplify platelet activation, aggregation and thrombin activation, and all three act in synergy to promote intravascular coagulation in sepsis (19, 38, 39). Evidence supporting the deleterious effects of NET-induced coagulation in infectious diseases will be discussed in the next section.

## Platelet-Driven NETosis

Unsurprisingly, platelet and neutrophil interactions are greatly increased during inflammatory responses. These interactions are, for the most part, initiated by soluble mediators, which directly activate these cells (Table 1). Co-incubation of healthy platelets and neutrophils with plasma from septic patients has been shown to promote platelet adhesion to neutrophils in a TLR4-dependent manner, a result similar to what is observed following co-incubation of platelets, neutrophils and LPS (34, 36). Moreover, LPS-induced intravascular NETosis and trapping of *Escherichia coli* in NETs has been shown to be augmented in the presence of platelets (34). Platelet-driven NETosis has been observed in the presence of all classic platelet agonists (i.e., thrombin, ADP, collagen, arachidonic acid) as well as several TLR ligands; however, in these models, NET formation does not occur in the absence of platelet activation (35–37). Hence, it is consensus that, for platelet-induced NETosis *in vitro*, platelets must be first activated. CD62P is also required for platelet-induced NETosis as CD62P^−/−^ platelets have been shown to fail to promote the release of NETs whereas overexpression of platelet CD62P enhanced phorbol 12-myristate 13-acetate (PMA)- and ionomycin-induced NETosis (35). The role of other surface molecules involved in platelet-neutrophil interactions, GPIb/IIa, CD11b (integrin α-chain L), and CD18 (integrin β-chain 2), remains debatable as some studies have shown these molecules to be dispensable (37) while other models have indicated a clear need for integrin-mediated platelet adhesion in the induction of NETosis (69). Although there is strong evidence that platelet-neutrophil adhesion plays a central role in platelet-induced NET formation, physical interactions between platelets and neutrophils may not be absolutely required as activated platelets are known to shed CD62P. Indeed, neutrophils, in the presence of *Streptococcus mutans* and soluble CD62P (sCD62P) have been shown to produce NETs, thus indicating that direct interactions are dispensable in some situations, at least *in vitro* (18).

**Table 1:** Platelet molecules that modulate neutrophil activation.

Factors stored in granules	**Adhesive glycoproteins:** P-selectin*(27, 40), Fibrinogen (41), vWF*(36, 42, 43), Fibronectin (44), Thrombospondin (44)
	**Coagulation factors:** Protein S (45), Factor XI (46)
	**Mitogenic factors:** PDGF (47), TGF-β (48), EGF (49)
	**Angiogenic / Vasoactive factors:** VEGF (50), PF4 inhibitor (51), Serotonin (52)
	**Chemokines:** CXCL7 (53), CXCL4*(PF4) (54, 55), CXCL1 (GROα) (56, 57), CXCL5*(58), CCL5*(RANTES) (59, 60), CCL3 (MIP1α) (61)
Unknown location	CCL7(MCP3) (56), IL1β (62), HMGB1*(63), Defensins*(11)
Plasma Membrane	Thromboxane A2* (36), PAF (64), CD40L (9), TREM-1 ligand (65), αIIbβb3 Integrin*(66, 67), GPIb*(36), ICAM2 (68), P-selectin*(35)

vWF, von Willebrand factor; PDGF, platelet derived growth factor; TGF-β, transforming growth factor β; VEGF, vascular endothelial growth factor; PF4, Platelet Factor 4; IL1β, interleukin 1β; HMGB1, high mobility group box protein 1; PAF, platelet activating factor; TREM-1, triggering receptor expressed on myeloid cells 1; GPIb, glycoprotein Ib; ICAM2, intercellular adhesion molecule 2. Factors known to be associated with NET production by neutrophils are denoted with an*.

In addition, to CD62P/PSGL-1 signaling, other mediators have been shown to be involved in triggering NET release. For instance, antibody-mediated blockade of platelet-derived high-mobility box group 1 protein (HMBG1) has been shown to inhibit NET formation *in vitro* (37). In fact, NET release was inhibited in the presence of anti-HMBG1 to a greater extent than in the presence of anti-CD62P. Moreover, HMGB1 was shown to be required for activation of autophagy pathways, which are required for NETosis in a RAGE-dependent manner, a key receptor for HMGB1 (37). A role for thromboxane A2 (TXA2) has also been demonstrated in platelet-driven NET release. In a study by Caudrillier et al. (66), activated, but not resting, platelets were shown to induce NET formation *in vitro*. This process was shown to be dependent on TXA2 receptor-mediated signaling, which activates the MAPK pathway (66). Moreover, platelet-derived β-defensin 1 was shown to induce NET formation in a ROS-dependent manner (70). Human platelets store β-defensin 1, an antimicrobial peptide primarily expressed by epithelial cells, in extragranular cytoplasmic compartments. Thus, β-defensin is not released during classic platelet degranulation stimulated by agonists such as thrombin or platelet activating factor (PAF). Instead, β-defensin 1 was shown to be released when platelets were stimulated in the presence of *Staphylococcus aureus*-derived α-toxin but not LPS (15). This α-toxin-platelet-β-defensin 1 axis could represent a novel mechanism by which platelets directly induce NETs in response to Gram-positive infections. The generation of NETs, however, was only demonstrated in the presence of purified β-defensin 1 (70). Co-incubation of α-toxin, platelets and neutrophils could provide more conclusive evidence of this putative interplay.

## Effects of NETs on Platelets and Coagulation

The downstream effects of platelet-induced NET release have also been studied. Activated, but not resting, platelets and neutrophils were shown to result in NET release and increased monolayer permeability in LPS-stimulated endothelial cells *in vitro* (66). Importantly, NETs also affect platelet function and direct evidence demonstrates that the platelet-NET axis is by no means a one-way road. In a report by Elaskalani et al. (38), cell-free NETs (collected from PMA-stimulated neutrophils) were incubated with human platelets where the presence of NETs alone (with no other platelet agonists added) was shown to promote platelet aggregation, secretion of ATP and ADP, and increased expression of CD62L and phosphatidylserine (PS) on the surface of the platelets (38). An increase in protein phosphorylation at tyrosine residues pointed to NET-mediated activation of intracellular signaling in platelets. Accordingly, Fuchs et al. (39) have demonstrated a role for DNA as well as histones H3 and H4 in NET-induced platelet aggregation *in vitro* (39). These results strongly hint at a positive feedback loop between platelets and NETs. NET-dependent platelet aggregation, however, was shown to be unaffected by DNase or heparin, suggesting mechanisms independent of the DNA scaffold and thrombin. Inhibition of cathepsin G, a NET component, reduced surface expression of CD62P and PS exposure in platelets whereas blockade of GPIIb/IIIa significantly inhibited platelet aggregation without affecting CD62P expression (38). These observations suggest multiple pathways are likely involved in NET-induced platelet activation and aggregation.

Furthermore, NETs have been implicated in directly inducing thrombin generation through both platelet-dependent and independent mechanisms. NETs released from PMA-stimulated neutrophils have been shown to increase thrombin generation in platelet-poor plasma. This activation required coagulation factors XII and XI, pointing to the involvement of the intrinsic coagulation pathway (71). Importantly, this mechanism was DNA-dependent, as thrombin generation was abrogated when DNase was added to the system (71, 72), although the exact role of DNA scaffold in this context is unknown. Given that coagulation factors optimally assemble on negatively-charged surfaces (such as the PS-rich plasma membrane of activated platelets and microparticles), and that DNA is a negatively-charged molecule, perhaps the DNA backbone of NETs serves as a somewhat ideal surface for the formation of coagulation factor complexes. Importantly, some studies have demonstrated a failure of purified NETs to induce thrombin generation. Whereas, the purified DNA backbone from NETs was able to induce thrombin generation in platelet-poor plasma, intact NETs, failed to generate active thrombin pointing out clear differences between *in vivo* and *in vitro* assays and suggesting qualitative differences in NETs themselves exist (73). In the presence of platelets, NETs were also shown to contribute to thrombin generation. The effect was dependent on platelet expression of TLR-2 and TLR-4, suggesting that NET components may act as receptor agonists, inducing platelet activation. Interestingly, DNase potentiated platelet- and NET-driven thrombin generation, likely because dismantling of NETs led to enhanced release of NET components, making them more readily available to act as platelet agonists (71).

In addition to NET production, PMA-stimulated neutrophils have also been shown to release microparticles, which attach themselves to NETs via PS residues. Blocking this interaction was implicated in reduced NET-dependent thrombin generation pointing to a role for neutrophil-derived microparticles in addition to DNA and other NET components (72). While these reports were largely done *in vitro*, the direct effect of NETs on coagulation *in vivo* has been also been investigated (19, 72). Using a model of sepsis following CLP, increased cell-free DNA in plasma was shown to be largely derived from neutrophils. Plasma levels of DNA-histone complexes were significantly inhibited in neutrophil-depleted animals, strongly suggesting that neutrophils undergo NETosis in the vasculature during sepsis. Thrombin-antithrombin (TAT) complex levels, a marker of thrombin generation, were increased 24 h after CLP. Moreover, thrombin generation *ex vivo* was decreased in these animals due to consumption of coagulation factors, a clinical manifestation of disseminated intravascular coagulation (DIC) in septic patients. In animals treated with DNase prior to CLP, thrombin generation *ex vivo* was restored, placing NETs as critical factor for the depletion of coagulation factors during sepsis (72). Additionally, blockade of NETs has been shown to reduce thrombin activity in the liver and lung microvasculature and inhibition of thrombin prevents NET-induced liver damage in an *in vivo* model of *E. coli*-induced sepsis (19), providing a direct link between NETs and coagulation. Interestingly, NETs have been shown to contribute to the occlusion of vessels in the lung microvasculature independently of thrombin generation in animals deficient for DNase1 and 3 during sepsis (74). These data indicate that NETs engage multiple mechanisms leading to microvascular obstruction, impairing organ perfusion and driving tissue damage.

Taken together, these studies have contributed greatly to mapping of the molecular mechanisms underlying platelet-NET interplay. While these mechanisms remain incompletely known, crucial molecules, such as CD62P and HMGB1 in platelets, PSGL1 in neutrophils and cathepsin G, histones and DNA in NETs, have been identified as potential targets for uncoupling immunity and coagulation. Targeting of these interactions, however, may have profound impact on host defense, underscoring the need for studying platelet-neutrophil crosstalk in *in vivo* models of infection and inflammatory disease.

## Targeting Platelet-NET Interactions in Infection-Induced Vascular Dysfunction

In support of experimental evidence of NET-induced coagulation, a correlation between NETs and thrombosis has been previously demonstrated in clinical studies (75–77). Various components of NETs (cell-free DNA, citrullinated H3 and nucleosome) were shown to be significantly increased following acute ischemic stroke. Levels of citrullinated H3 were also associated with mortality at the 1-year follow up assessment of the study, which included over 200 patients with acute ischemic stroke (75). Of note, H3 citrullination weakens histone binding to negatively charged DNA, which in turn favors chromatin decondensation, a critical step for NETosis (78). Furthermore, a descriptive study of the composition of thrombi retrieved from ischemic stroke patients has revealed the presence of activated neutrophils (CD66b+ and neutrophil elastase+ granulocytes) and as well as NETs (extracellular DNA with citrullinated H3) (76). In the context of infectious diseases, a study by Yang et al. (77) has compared the ability of neutrophils from septic patients to undergo NETosis and its implications in thrombin and fibrin generation (77). Neutrophils from septic patients were able to promote thrombin and fibrin generation in the presence of control plasma in a DNA-dependent manner. Thus, NET release alone was associated with risk of thromboembolism. However, it is still unclear if NETs are causative of thrombosis or if NETosis is a consequence of thrombus formation.

Clinical evidence has supported the role of NETs in driving coagulation and ultimately, vascular dysfunction in both primarily cardiovascular disorders (i.e., ischemic stroke) as well as conditions initiated by infections, such as sepsis. These reports, however, did not delve into the implications of disrupting NET-induced coagulation for disease outcome. Perhaps the main concern when considering this strategy is the impairment of the innate immune response. To address this issue, animal models of systemic or highly invasive infections have been very insightful (Figure 1B). Infective endocarditis is a condition involving thrombi formation following an immune response to bacterial colonization in the heart. Vegetations, a pathological feature of endocarditis, are composed of bacteria embedded in a mass of platelets, fibrin, and immune cells, such as neutrophils. In a rat model of *Streptococcus mutans*-induced endocarditis, NETs were identified in vegetations on damaged heart valves (18). Accordingly, pre-treatment with DNase I, which dismantles the NET web-like structure, led to a decrease in vegetation weight and bacterial load. However, the effect was also accompanied by increased bacterial dissemination. These results suggest a protective role of thrombus and NET formation on heart valves, at least in restricting pathogen invasion and dissemination throughout the body. Indeed, *in vitro*, in the presence of neutrophils, platelets were shown to form aggregates around bacteria. The effect was abrogated in the presence of DNase I, supporting the involvement of NETs in pathogen trapping (18).

Furthermore, in models of bacterial sepsis, NET formation, platelet aggregation and thrombin activity were associated with impaired perfusion of the liver microvasculature and organ damage. PAD4^−/−^ mice (unable to release NETs from neutrophils) or pre-treatment with DNase I markedly reduced thrombin activity, suggesting that NETs directly contributed to thrombin activation, likely through platelet-dependent mechanisms. Moreover, organ perfusion and function were improved in the absence of NETs, suggesting that direct targeting of NETs may be beneficial in the context of disseminated infections characterized by overt inflammation, such as sepsis (19). Importantly, this beneficial effect of NET prevention/dismantlement is very much context dependent. In a study by Lefrançais et al. (20), NETs, as expected, were associated with increased inflammation, lung damage and early mortality in a model of bacteria-induced lung injury (20). However, NET-deficient mice or removal of NETs with DNAse I, was associated with increased bacterial loads, demonstrating a clear role for NETs in restricting pathogen dissemination. Critically, the survival rate of animals in this model was only improved if NETs were targeted at earlier time points post-infection. At later time points (>40 h from the i.t., administration of *S. aureus*), blockade of NETs was no longer protective. These results suggest that although NETs play a role in immunopathology early in infection, their role in trapping and sequestering microorganisms is also critical to limit dissemination of infection and as such, complete abrogation of NET production may be just as deleterious to the host as NET-induced pathology.

## Summary

Platelet-neutrophil interactions are undoubtedly a two-way relationship due, in large part, to the role of NETs in modulating both platelet and neutrophil function. Moreover, clinical and experimental studies have supported that NET-mediated coagulation and immunity are critical to disease outcome. Perhaps not surprisingly, NETs seem to play a dual role in models of infectious diseases: they orchestrate both immunopathology and infection clearance. Interestingly, the direct participation of NETs in amplifying coagulation through platelet activation, thrombin generation and microparticle release, seems to ultimately underlie NET-induced immunopathology. Importantly, while blocking or dismantling NETs ameliorates coagulation dysfunction, it may also impair pathogen clearance. Although separating immunity and coagulopathy is the ultimate goal, the question still remains… is this a matter of simply fine tuning of platelet-neutrophil interactions, or, is targeting the procoagulant components of NETs the key to this therapeutic avenue? In the end, a more thorough understanding of the molecular mechanisms underlying NET-driven coagulation will be needed if we are to uncouple immunity and coagulation in the setting of infectious disease.

## Author Contributions

AZ and CJ contributed to manuscript generation and revision and read and approved the submitted version.

### Conflict of Interest Statement

The authors declare that the research was conducted in the absence of any commercial or financial relationships that could be construed as a potential conflict of interest.
